# Enhancing ECG Classification Generalization Through Unified Multi-Dataset Training

**DOI:** 10.3390/s26061830

**Published:** 2026-03-13

**Authors:** Minchan Kim, Miyoung Shin

**Affiliations:** Bio-Intelligence & Data Mining Laboratory, School of Electronic and Electrical Engineering, Kyungpook National University, Daegu 41566, Republic of Korea; dies53166@naver.com

**Keywords:** atrial fibrillation, electrocardiogram, contrastive learning, representation learning, multi-dataset training, artificial intelligence

## Abstract

Atrial fibrillation (AF) is one of the most prevalent and clinically significant cardiac arrhythmias, and electrocardiography (ECG) is widely used for its detection. However, existing models often exhibit performance degradation when applied to unseen data due to dataset-specific biases and distributional shifts. This limited generalization remains a major obstacle to reliable clinical deployment. To address this, we propose a multi-dataset ECG classification framework designed to improve cross-dataset robustness. The model employs supervised contrastive learning and layer-wise normalization to stabilize training and mitigate the influence of domain-specific variations. The proposed approach was evaluated under a Leave-One-Dataset-Out setting, achieving an average *accuracy* of 97.5% and an *F1-score* of 89.3%. It consistently demonstrated superior performance compared with single-dataset training and naïve multi-dataset aggregation. These results indicate that the proposed framework can contribute to more stable automated AF detection across diverse clinical environments.

## 1. Introduction

Atrial fibrillation (AF) is a cardiac arrhythmia characterized by rapid and irregular electrical activity in the atria, leading to irregular heart rhythms. It is one of the most common cardiovascular disorders and is closely associated with serious complications such as stroke and heart failure [1,2]. Patients with AF have been reported to have a substantially higher risk of stroke compared with the individuals without AF [3]. Therefore, the accurate identification of AF has become increasingly important in clinical practice.

Electrocardiography (ECG) is a primary diagnostic tool for the detection and monitoring of AF [4]. ECG is widely used not only in hospital environments but also in wearable devices because of its non-invasive nature and cost-effectiveness. In recent years, this widespread use has motivated extensive studies on automated ECG-based rhythm classification using machine learning [5,6] and deep learning approaches [7,8,9,10].

Despite growing research efforts in ECG classification, several challenges remain in real-world clinical settings. Deep learning approaches typically require large-scale and accurately annotated ECG datasets [11], as ECG annotation is time-consuming, labor-intensive, and requires expert knowledge [12,13]. Publicly available ECG datasets, therefore, are often limited in both size and diversity, with individual datasets representing only a restricted range of clinical conditions and patient populations. This limitation has motivated increasing interest in utilizing multiple ECG datasets [11].

**Figure 1:**
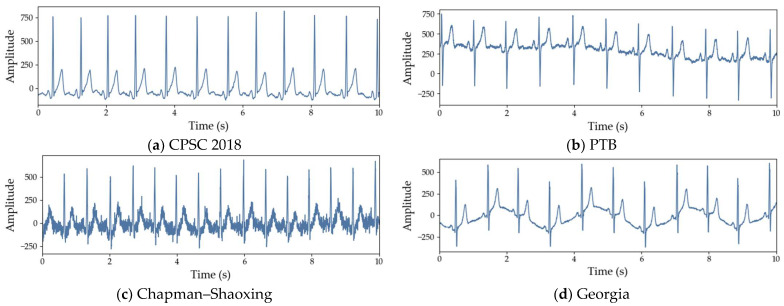
Normal ECG waveform examples from different datasets: (**a**) CPSC 2018; (**b**) PTB; (**c**) Chapman–Shaoxing; (**d**) Georgia.

However, effectively utilizing multiple ECG datasets is non-trivial [14]. ECG signals inherently exhibit high variability due to differences in recording devices, acquisition environments, and patient characteristics [15]. As illustrated in Figure 1, even ECG signals corresponding to the same normal rhythm can differ markedly when drawn from different datasets. Such inter-dataset differences may lead models to overfit dataset-specific characteristics, thereby degrading performance on unseen datasets [16,17].

To mitigate inter-dataset variability, domain adaptation approaches incorporate information from the target dataset during training to reduce discrepancies between source and target data distributions. Among these approaches, adversarial learning has been widely used to suppress dataset-dependent information in learned representations [18,19,20]. Some approaches additionally model both dataset-specific and invariant features to better capture heterogeneous characteristics across datasets [21].

In practice, data from the target dataset are often unavailable during training. Domain generalization addresses this setting by learning representations that generalize across source datasets without access to target-dataset information. Adversarial learning combined with feature synthesis has been explored to introduce artificial distributional variations during training [22]. Multi-level representation learning has also been investigated, aggregating features from different network depths to capture ECG characteristics at multiple abstraction levels [23]. In addition, progressive deep learning frameworks combine time–frequency representations with complementary hand-crafted features to improve robustness and efficiency [24].

However, learning dataset-invariant ECG representations still remains challenging. Dataset-specific biases often distort rhythm patterns, making it difficult for models to capture consistent features across different data sources. Therefore, a learning strategy is required to explicitly align representations of the same rhythm class while suppressing irrelevant variations. To this end, we employ supervised contrastive learning to encourage ECG signals from the same rhythm class to form consistent representations in the feature space. This learning strategy guides the model to focus on rhythm-relevant characteristics instead of dataset-specific statistical patterns.

To jointly support representation learning and rhythm classification, we employ a dual-head architecture composed of a projection head and a classification head. Both heads share a common feature extractor and are optimized using supervised contrastive loss and classification loss, respectively. This design enables the model to learn representations that balance class-level alignment and discriminative capability within a unified feature space.

In addition, a layer-wise normalization strategy is incorporated into the feature extractor to reduce sensitivity to inter-dataset distribution differences. Batch normalization is applied in the early layers, while instance normalization is used in deeper layers. This configuration helps suppress dataset-dependent variations while preserving essential information for rhythm classification.

The objective of this study is to improve ECG classification across diverse datasets. We focus on capturing consistent rhythm features regardless of data variations.

The main contributions of our work are summarized as follows:

1. We introduce a supervised contrastive learning framework specifically designed for multi-dataset ECG classification. This approach ensures that the model captures consistent rhythm features across different databases by learning a feature representation.
2. We propose a dual-head architecture that effectively balances representation learning with arrhythmia classification. This design enables the feature extractor to learn a discriminative and well-structured feature space without the need for complex or heavy model architectures.
3. We implement a layer-wise normalization strategy using instance normalization and batch normalization to stabilize the feature distribution across diverse data conditions. This strategy suppresses dataset-specific biases in the feature space, ensuring that the learned representations remain robust to inter-dataset distribution shifts.
4. We demonstrate that our framework achieves robust generalization with significantly higher stability across unseen datasets. By minimizing the performance variance between different datasets, we confirm that the learned feature representations provide a more reliable foundation for deployment in realistic clinical scenarios.

## 2. Materials and Methods

### 2.1. Datasets

To evaluate the generalization performance of the proposed model, five publicly available ECG datasets were used in this study: the Chapman University–Shaoxing People’s Hospital ECG Database (Chapman–Shaoxing) [25], the Georgia 12-Lead ECG Challenge Database (Georgia), the China Physiological Signal Challenge 2018 Database (CPSC 2018) [26], the Physikalisch-Technische Bundesanstalt Diagnostic ECG Database (PTB) [27], and PTB-XL [28]. These datasets were selected to reflect diverse acquisition conditions, recording protocols, and patient populations.

In this study, a binary classification task distinguishing normal and AF rhythms was considered. These two classes were selected because they are consistently represented across all five datasets, providing sufficient samples for a robust evaluation of cross-database generalization. Following the standardized labeling conventions of the PhysioNet challenges, recordings were categorized based on their assigned SNOMED CT codes [29]. To ensure label consistency, only records with a single rhythm annotation were included, while other arrhythmia types and multi-labeled records were excluded.

The main characteristics of each dataset are summarized below:

1. Chapman–Shaoxing was jointly developed by Chapman University and Shaoxing People’s Hospital in China. It includes 45,152 ECG recordings, all of which are 10 s in duration and sampled at 500 Hz.
2. CPSC 2018 was released as part of the China Physiological Signal Challenge held in Nanjing, China. It contains 13,256 ECG recordings with durations ranging from 6 s to 144 s, all recorded at a sampling frequency of 500 Hz.
3. Georgia was constructed primarily at Emory University and represents ECG data from a population in the southeastern United States. It contains 20,672 ECG recordings with durations between 5 s and 10 s, recorded at a sampling frequency of 500 Hz. The data were collected under conditions representative of routine clinical practice.
4. PTB is a clinical ECG dataset collected in Germany and includes 549 recordings from 290 subjects. Each recording consists of 15 signals, including the standard 12-lead ECG and Frank leads, with a baseline sampling frequency of 1000 Hz. The dataset also provides detailed clinical metadata such as age, sex, and diagnostic information.
5. PTB-XL is a large-scale clinical ECG dataset comprising 21,837 12-lead recordings collected from 18,885 patients. All recordings are 10 s in duration and sampled at 500 Hz. Each record includes diagnostic, form, and rhythm information following the SCP-ECG standard.

An overview of the datasets, including their sources, number of subjects, number of records, recording lengths, and sampling frequencies, is provided in Table 1.

### 2.2. Data Preprocessing

In this study, lead I was used for all experiments. Lead I is commonly available in wearable devices and provides sufficient information for rhythm classification [30,31].

All ECG signals were resampled to 500 Hz for consistency. These signals were then divided into non-overlapping 10 s segments, resulting in a fixed length of 5000 time points per segment [32,33]. This duration was chosen to provide a sufficient number of heartbeats to reliably analyze beat-to-beat intervals while ensuring a consistent input dimension. Recordings shorter than 10 s were excluded to maintain data integrity. After segmentation, the numbers of normal and AF samples used in the experiments for each dataset are summarized in Table 2.

**Figure 2:**
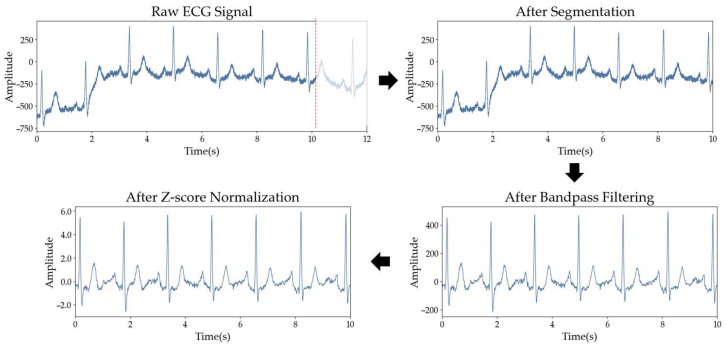
ECG preprocessing steps applied in this study, including segmentation, bandpass filtering, and z-score normalization.

To eliminate artifacts while preserving clinical features, a fourth-order Butterworth bandpass filter with a passband of 1–45 Hz was applied [34]. This range was selected to effectively suppress low-frequency baseline wander caused by respiration and high-frequency powerline interference, without distorting the essential QRS complex morphology. Score normalization was applied to each ECG segment to standardize signal amplitudes by centering the data at a zero mean with unit variance. This normalization prevents the model from being biased by extreme amplitude peaks, ensuring that the overall morphological patterns are preserved regardless of fluctuations or signal outliers. Figure 2 depicts the overall ECG preprocessing pipeline.

### 2.3. Model Architecture

**Figure 3:**
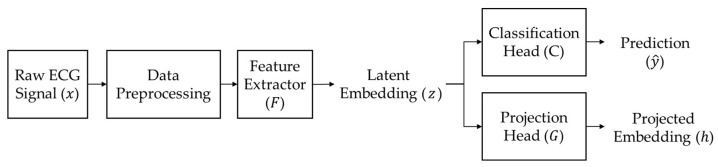
Overview of the proposed model architecture for multi-dataset ECG rhythm classification.

Figure 3 illustrates the overall architecture and processing flow of the proposed model. The model takes a preprocessed single-lead ECG signal of 10 s, denoted as x∈X, as input. The feature extractor $F_{\theta }:X\to Z\;$ extracts a latent embedding ${z=F}_{\theta }\left(x\right)\;$ from the input ECG signal. The extractor is designed with a layer-wise normalization strategy, where different normalization schemes are applied at different network depths.

The extracted latent embedding z is subsequently fed into two heads. The classification head $C_{\psi }:Z\to Y\;$ produces the final rhythm prediction between normal and AF based on the latent representation. The projection head $G_{\phi }:Z\to H\;$ maps the latent embedding into a separate feature space used for supervised contrastive learning.

The feature extractor and both heads are jointly optimized using the classification and contrastive objectives during training. During inference, only the feature extractor and the classification head are used to generate the final prediction for an input ECG signal.

#### 2.3.1. ECG Feature Extractor with Layer-Wise Normalization

The feature extractor is designed to learn discriminative and generalizable representations for rhythm classification across heterogeneous ECG datasets. It takes a single-lead ECG signal of 10 s, represented as $x\in R^{1\times 5000},$ as input. The input signal first passes through an initial convolutional layer, followed by batch normalization, a ReLU activation function, and a max pooling layer. The network then consists of a total of eight residual blocks.

The feature extractor follows a ResNet-based architecture with layer-wise normalization applied across different depths. Unlike standard ResNet configurations that apply a single normalization strategy throughout the network, different normalization schemes are applied at different network depths to better handle inter-dataset variability.

**Figure 4:**
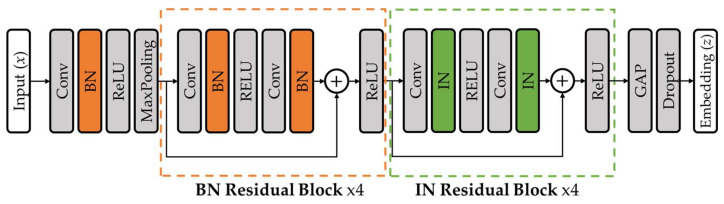
Architecture of the ECG feature extractor with layer-wise normalization. Orange boxes represent batch normalization, green boxes represent instance normalization, and dashed lines indicate the repeated block structures.

Batch normalization is applied to the initial convolutional layer and the first four residual blocks. This design stabilizes mini-batch activation distributions in early layers, facilitating robust low-level feature extraction under multi-dataset training. Instance normalization is applied to the remaining four residual blocks. By normalizing activation statistics independently for each sample, this strategy reduces the influence of dataset-specific distributional differences on higher-level representations. The transition point was determined empirically to achieve an optimal trade-off between preserving low-level structural features and maintaining high-level style invariance. This approach filters out dataset-specific stylistic noise while preserving the essential morphology of the signals, which has been shown to improve generalization performance [35]. Figure 4 illustrates the overall architecture of the proposed ECG feature extractor.

#### 2.3.2. Dual-Head Architecture

A dual-head architecture, consisting of a projection head and a classification head, is designed to jointly perform supervised contrastive learning and rhythm classification. The latent embedding *z* extracted by the feature extractor is fed into two parallel heads, each optimized for a different learning objective.

The classification head produces the final prediction for binary rhythm classification. It consists of two fully connected layers and outputs logits for rhythm classification, which are optimized with a binary cross-entropy loss. The projection head maps the latent embedding into a lower-dimensional embedding space. It is also implemented using two fully connected layers and outputs embeddings for supervised contrastive loss computation.

By adopting this dual-head design, the feature extractor is encouraged to learn representations that are simultaneously discriminative for rhythm classification and well-structured for contrastive representation learning.

The complete specifications of the integrated architecture, covering the entire pipeline from the feature extractor to both heads, are provided in Appendix A.

### 2.4. Training Strategy

To increase the diversity of sample relationships for supervised contrastive learning, each training mini-batch is constructed using ECG samples drawn from multiple datasets. Let a mini-batch be defined as

(1) $$B=\{(x_{i},\;y_{i},\;d){\}}_{i=1}^{N},$$

where $x_{i}\in X\;$ denotes an input ECG segment, $y_{i}\in \{0,\;1\}$ is the corresponding rhythm label, and $y_{i}\in D$ indicates the dataset from which the sample is drawn. To ensure robust cross-dataset representation learning, each mini-batch is constructed such that at least one Normal and one AF sample are included. This batch composition allows samples with the same or different rhythm labels to be compared across datasets.

Each input ECG signal $x_{i}$ is first mapped to a latent embedding through the feature extractor $F_{\theta }$ as

(2) $$z_{i}{=F}_{\theta }\left(x\right).$$

The resulting latent embedding $z_{i}$ is then forwarded to two heads for rhythm classification and supervised contrastive learning.

The classification head $C_{\psi }$ takes $z_{i}$ as input and produces a prediction for the rhythm label. The output is converted into a probability ${\hat{y}}_{i}$ for the AF class, and the classification loss is defined using the binary cross-entropy loss,

(3) $$L_{BCE}=-\frac{1}{N}\sum_{i=1}^{N}\left[y_{i}\log\left({\hat{y}}_{i}\right)+\left(1-y_{i}\right)\log\left(1-{\hat{y}}_{i}\right)\right],$$

where ${\hat{y}}_{i}=C_{\psi }(z_{i})$. This loss promotes discriminative feature learning for binary rhythm classification.

The projection head $G_{\phi }$ maps the latent embedding into a lower-dimensional feature space for contrastive learning,

(4) $$h_{i}=G_{\phi }\left(z_{i}\right),\;\;\;|\left|h_{i}\right|{|}_{2}=1.$$

Samples sharing the same rhythm label are treated as positive pairs, while samples with different labels are treated as negative pairs. The supervised contrastive loss is defined as

(5) $$L_{SupCon}=\sum_{i}\frac{-1}{\left|P(i)\right|}\sum_{p\in P(i)}log\frac{exp(h_{i}\cdot h_{p}/\tau )}{\sum_{a\in A(i)}exp(h_{i}\cdot h_{a}/\tau )},$$

where P(i) denotes the set of positive samples sharing the same label as sample i, A(i) represents all samples within the mini-batch, and *τ* is a temperature parameter.

The overall training objective is defined as a weighted combination of the classification loss and the supervised contrastive loss,

(6) $$L_{Total}=\alpha L_{BCE}+\left(1-\alpha \right)L_{SupCon},$$

where α∈[0,1] controls the relative contribution of the two loss terms.

The training process is summarized in Algorithm 1.
**Algorithm 1:** Supervised contrastive training for ECG arrhythmia classification1:This algorithm outlines the training procedure for multi-dataset ECG classification using supervised contrastive learning.
**Input**: Multi-dataset ECG samples $\left\{\left(x_{i},\;y_{i},\;d_{i}\right)\right\}$ from,       feature extractor $F_{\theta }$, classification head $C_{\psi }$, projection head $G_{\phi }$,
      loss weight α, temperature parameter τ, training epochs E

**Output**: Trained parameters θ, ψ,ϕ2:**Initialize** parameters θ, ψ,ϕ
3:**for** epoch = 1 **to**
*E*
**do**4:    **for** each min-batch **do**5:        Sample a mini-batch6:        $B=\{\left(x_{i},\;y_{i},\;d_{i}\right){\}}_{i=1}^{N}$ from multiple datasets7:        **Subject to** $\forall k\in \{d_{i}{\}}_{i\in B},\;\forall c\in \left\{Normal,\;\;AF\right\}\;:\;\sum_{i\in B}1(y_{i}=c,d_{i}=k)\geq 1$8:        **for each** $(x_{i},\;y_{i})\in B$
**do**9:      Compute latent embedding10:      $z_{i}=F_{\theta }\left(x\right)$
11:      Compute projection embedding12:      $h_{i}=G_{\phi }\left(z_{i}\right),\;\;\;\;\;\;h_{i}\leftarrow h_{i}/|\left|h_{i}\right|{|}_{2}$
13:      Compute classification prediction14:      ${\hat{y}}_{i}=C_{\psi }(z_{i})$
15:        **end for**
16:        Compute binary cross-entropy loss $L_{BCE}$ using Equation (3)17:        Compute supervised contrastive loss $L_{SupCon}$ using Equation (5)18:        Compute total loss19:        $L_{Total}=\alpha L_{BCE}+(1-\alpha )L_{SupCon\;}$
20:        Update θ, ψ,ϕ by backpropagation21:    **end for**
22:**end for**

## 3. Results

### 3.1. Experimental Setup

The overall training objective for ECG classification was a combination of two loss terms, $L_{SupCon}$ and $L_{BCE}$. The balancing weight α between the two losses was set to 0.5 for all experiments. The temperature parameter τ used in supervised contrastive learning was fixed to 0.07. The batch size was set to 64. The dimensions for the latent and projected embeddings were set to 512 and 32, respectively. The rationale for selecting these specific parameter values is further analyzed in Section 3.5.2 and the Appendix B.

Model training was conducted under a Leave-One-Dataset-Out (LODO) evaluation protocol [36]. In each experiment, one dataset was held out as the test set, while the remaining datasets were used for training and validation. This setting was adopted to evaluate the ability of the model to generalize to unseen ECG dataset.

From the training data, 10% of the samples were randomly selected as a validation set. Early stopping was applied based on the validation loss, and training was terminated if no improvement was observed for five consecutive epochs. The maximum number of training epochs was set to 50. The Adam optimizer was used for all experiments with parameters β_1_ = 0.9 and β_2_ = 0.999. The initial learning rate was set to 1 × 10^−3^. We employed an early stopping mechanism with a patience of five epochs and a ReduceLROnPlateau scheduler, which halved the learning rate if validation loss stagnated for two consecutive epochs.

All experiments were implemented using PyTorch 2.5 and executed on an NVIDIA GeForce RTX 4060 Ti GPU environment with CUDA 12.1. Detailed computational requirements are provided in Table A3 of Appendix A.

### 3.2. Evaluation Metrics

The classification performance of the proposed model was evaluated using *accuracy*, *precision*, *recall*, *specificity*, *F1-score*, *AUC- ROC* and *PR-AUC*.

*Accuracy* measures the proportion of correctly classified samples among all samples and is defined as

(7) $$Accuracy=\frac{TP+TN}{TP+TN+FP+FN}$$

where *TP* and *TN* denote the numbers of correctly classified normal and AF samples, respectively, and *FP* and *FN* represent false positives and false negatives.

*Precision* is the proportion of positive identifications that were actually correct, reflecting the model’s predictive *accuracy* for the positive class:

(8) $$Precision=\frac{TP}{TP+FP}\;$$

*Recall*, also referred to as sensitivity, measures the proportion of actual positives that were correctly identified, indicating the model’s ability to detect the positive class:

(9) $$Recall=\frac{TP}{TP+FN}\;$$

*Specificity* was employed to evaluate the model’s ability to correctly identify the negative class. It is defined as the proportion of actual negatives that are correctly identified as such:

(10) $$Specificity=\frac{TN}{TN+FP}\;$$

To account for class imbalance, the *F1-score* was also used. The *F1-score* is defined as the harmonic mean of *precision* and *recall*,

(11) $$F1-score=\frac{2\times Precision\times Recall}{Precision+Recall}\;$$

The *ROC-AUC* represents the area under the receiver operating characteristic curve, which illustrates the relationship between the true positive rate and the false positive rate across different decision thresholds. It reflects the overall discriminative capability of the model.

The *PR-AUC*, defined as the area under the *precision–recall* curve, was additionally considered. This metric is particularly informative in imbalanced classification settings, as it more sensitively captures the detection performance for the positive class.

### 3.3. Generalization Performance for ECG Classification

Table 3 compares the generalization performance of the proposed method with single-dataset training and a multi-dataset baseline. The baseline is trained by naively aggregating multiple datasets without explicitly addressing inter-dataset variability.

The proposed method for multi-dataset training consistently outperformed both single-dataset training and the multi-dataset baseline on each test dataset, achieving an average *accuracy* of 97.5% and an *F1-score* of 89.3%. In addition, the proposed method yielded strong performance in terms of *specificity* and *PR-AUC*, with average values of 96.2% and 92.8%, respectively.

When Chapman–Shaoxing was used as the test dataset, the proposed method achieved an *F1-score* of 97.7%, which corresponds to an improvement of 25.9% compared to single-dataset training and 31.4% compared to the baseline. On the PTB-XL test dataset, the proposed method recorded an *F1-score* of 72.9%, surpassing both single-dataset training (*F1-score*: 67.2%) and the baseline (*F1-score*: 58.0%).

The proposed method also substantially reduced performance variability across test datasets. This indicates that the proposed framework not only improves overall classification performance but also provides more consistent generalization across heterogeneous ECG datasets.

### 3.4. Characterization of Learned Representation Space

**Figure 5:**
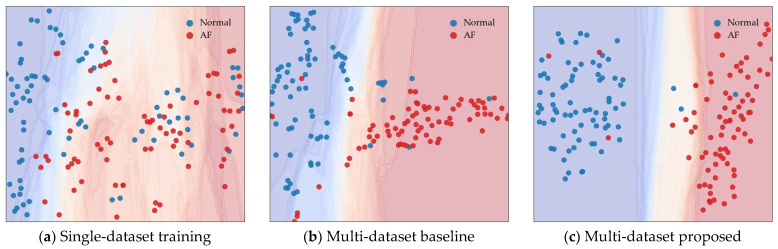
Comparison of sample distributions and decision boundaries in the representation space for different training strategies: (**a**) single-dataset training, (**b**) multi-dataset baseline, and (**c**) the proposed method for multi-dataset training.

Figure 5 illustrates the learned representation space formed under different training strategies. The decision boundaries are defined by classifiers trained on the corresponding training data, while the test samples are projected onto the same feature space. Each point represents an individual test sample, and the background color indicates the class-specific decision regions.

As shown in Figure 5a, when the model is trained on a single dataset, normal and AF samples are widely scattered and heavily overlapping in the representation space. As a result, the decision boundary between the two classes is poorly defined.

As shown in Figure 5b, the multi-dataset baseline achieves partial improvement in class separation compared to single-dataset training. However, a substantial number of test samples remain close to the decision boundary, indicating unstable class separation in the learned representation space.

In contrast, Figure 5c shows that the proposed method produces a more structured representation space. Samples belonging to the same rhythm class form more compact clusters and are separated by a clearer decision boundary. This separation is preserved for test samples collected from a different dataset, suggesting that the learned representations emphasize rhythm-relevant characteristics rather than dataset-specific variations.

### 3.5. Ablation Studies

#### 3.5.1. Effect of Layer-Wise Normalization

Table 4 presents a comparison of classification performance obtained by applying different normalization strategies within the feature extractor. Compared to configurations applying uniform batch or instance normalization across all layers, the proposed layer-wise normalization strategy achieved superior performance, reaching an *accuracy* of 97.5% and an *F1-score* of 89.3%.

**Figure 6:**
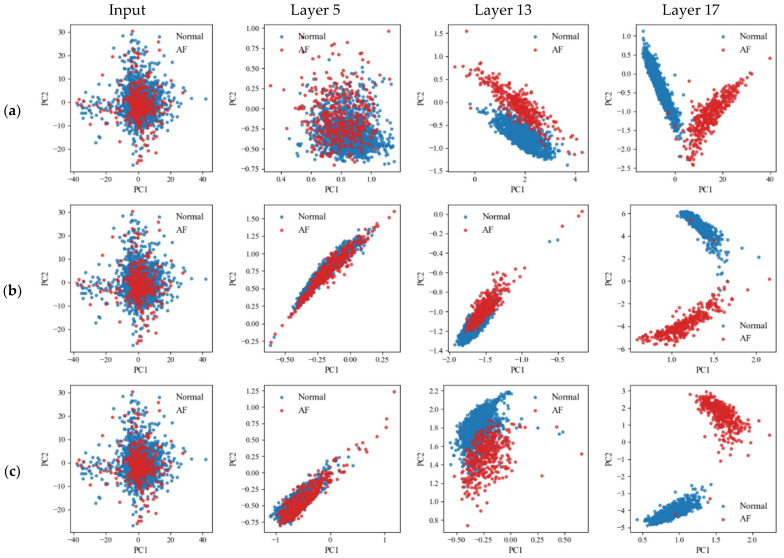
Feature space distributions across feature extractor layers for different normalization strategies: (**a**) Batch normalization; (**b**) Instance normalization; (**c**) Proposed layer-wise normalization.

Figure 6 presents the class distributions in the learned feature space at different network depths for each normalization strategy. With batch normalization applied across all layers, Normal and AF samples remain closely distributed even at the final layer (layer 17), showing limited class separation. When instance normalization is applied across all layers, separation between the two classes emerges only in deeper layers and remains relatively weak in early and intermediate layers.

On the other hand, with the proposed layer-wise normalization strategy, class separation becomes progressively clearer across network depth. The final layer demonstrates the most distinct separation compared with other normalization strategies. These observations support that the proposed normalization strategy promotes stable representation learning across network depth and yields more discriminative features for rhythm classification.

#### 3.5.2. Effect of Loss Function Configuration

Table 5 summarizes the classification performance under different loss function configurations. When the model was trained using only the supervised contrastive loss ($L_{SupCon}$), the *F1-score* was limited to 39.1% and the *accuracy* dropped to 63.9%. Training with only the binary cross-entropy loss ($L_{BCE}$) resulted in an *F1-score* of 75.2% and an *accuracy* of 88.1%, but performance gains remained constrained under the multi-dataset setting.

In contrast, the proposed loss formulation combining $L_{SupCon}$ and $L_{BCE}$ consistently achieved superior performance across evaluation metrics. With α = 0.25, the highest *specificity* of 98.7% was obtained, along with an *accuracy* of 92.9%. When α was set to 0.5, the model achieved an *accuracy* of 97.5% and a *F1-score* of 89.3%.

These results suggest that the two loss functions play complementary roles. While $L_{BCE}$ supports effective rhythm discrimination, $L_{SupCon}$ helps reduce inter-dataset variability by encouraging consistent representations across datasets.

### 3.6. Comparison with Existing Methods

Comparative experiments were conducted to evaluate the performance of the proposed method against existing approaches designed for multi-dataset learning. In addition to the naïve baseline model, representative methods were considered:

1. DANN (domain adversarial neural network) combines a rhythm classifier and a dataset discriminator within a shared feature extractor [18]. During training, the two objectives are optimized in an adversarial manner to encourage dataset-invariant feature representations.
2. DSBN (domain-specific batch normalization) shares convolutional layer weights across datasets while maintaining separate batch normalization statistics for each dataset, allowing dataset-dependent feature normalization [37].
3. MS-DANN (multi-scale domain adversarial neural network) is a modified architecture inspired by [38], which combines multi-scaled residual blocks for temporal feature extraction with a domain adversarial training strategy to enhance generalization.

As shown in Table 6, the adversarial domain adaptation method, DANN, achieved an *F1-score* of 75.5% and a *PR-AUC* of 91.4%. The DSBN approach improved performance over the baseline on several metrics, achieving an *F1-score* of 86.3% and a *PR-AUC* of 92.9%, which was the highest among the comparison methods. Additionally, MS-DANN reached an *F1-score* of 85.0% and a *PR-AUC* of 92.5%.

The proposed method achieved the best overall performance, with an *accuracy* of 97.5% and an F1-score of 89.3%. It outperformed all comparison methods in terms of *accuracy* and F1-score, while achieving a *PR-AUC* (92.8%) comparable to that of DSBN.

Table 7 compares the proposed framework with existing studies on AF classification using multiple ECG datasets. The proposed approach demonstrates competitive performance and stable generalization across diverse datasets using a unified training strategy with a single-lead ECG input.

### 3.7. Analysis of Class-Discriminative Activation Regions

**Figure 7:**
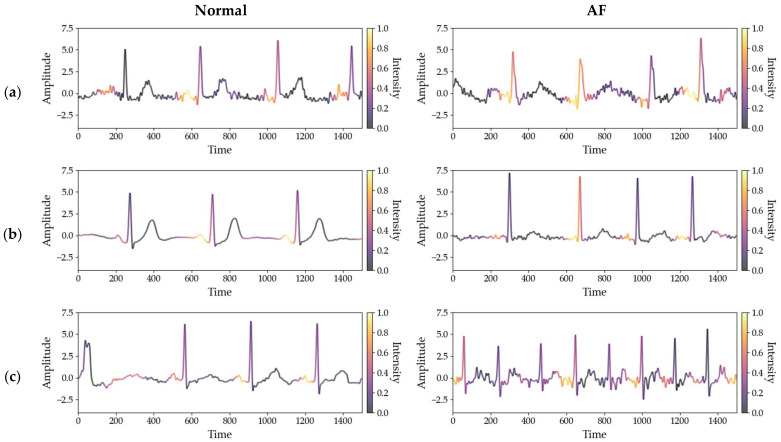
Class-discriminative attention patterns for normal and AF rhythms across different ECG datasets: (**a**) Chapman–Shaoxing; (**b**) CPSC 2018; (**c**) PTB-XL.

Figure 7 presents Grad-CAM visualizations to analyze whether the model consistently attends to rhythm-relevant ECG regions across different datasets. For normal rhythms, the model primarily attends to regions around the P-wave.

For AF rhythms, attention is mainly concentrated around the R-peaks and regions exhibiting abnormalities in the P-wave. These attention patterns are consistent with ECG features that are clinically used for AF detection [44].

Similar attention patterns are observed across datasets despite differences in signal characteristics and acquisition conditions. This indicates that the model relies on rhythm-relevant features rather than dataset-specific characteristics.

## 4. Discussion

In this study, we addressed the generalization problem from two perspectives: model architecture and normalization strategy. Our goal was to ensure the network extracts features that capture essential rhythm patterns rather than dataset-specific characteristics.

To achieve this, we first utilized supervised contrastive learning. The objective was to encourage ECG segments with the same rhythm to form consistent representations in the feature space, regardless of their origin. This approach allows the feature extractor to prioritize rhythm-related information over variations between different datasets.

Next, we implemented a layer-wise normalization strategy to address the limitations of standard methods. We observed that using only batch normalization often results in features where sample differences are heavily influenced by the specific dataset. To suppress these variations, we integrated instance normalization with batch normalization. This hybrid approach enables the model to reduce the influence of dataset-specific traits and focus on invariant rhythm patterns.

Overall, our results showed that the proposed framework achieved consistent performance improvements across all test datasets, regardless of the origin of the data. This stability across different test sets is a key outcome of our study. In particular, we observed significant gains in AF detection. AF is characterized by irregular rhythms, which typically leads to large performance variances between different datasets. Our framework effectively reduced this gap by capturing more robust features that are less sensitive to these variations.

However, this study has several limitations. To use rhythms that are commonly available across multiple diverse datasets, our scope was restricted to binary classification. This must be expanded to multi-class arrhythmia detection in the future.

Furthermore, performance remains suboptimal in certain cases. As shown in the confusion matrices in Appendix C, some normal waveforms are still misclassified as AF, likely because specific morphological features in those recordings are easily confused with arrhythmic patterns. There is also a concern regarding the segmentation process. While the original records are fully annotated, the process of cutting the entire record into segments might not have captured enough of the representative rhythm in some cases.

Finally, we observed that adding more datasets does not always yield significant improvements. In cases where performance is already high, merging additional data can sometimes have a limited impact on the learning process. This suggests that future work should explore more selective dataset integration strategies to ensure optimal learning.

## 5. Conclusions

This study addresses the cross-dataset generalization problem in multi-dataset ECG classification by reducing the model’s reliance on dataset-specific characteristics. Training deep learning models on multiple ECG datasets often leads to overfitting to dataset-specific patterns, resulting in degraded performance on unseen datasets. In contrast, the proposed framework emphasizes rhythm-discriminative representations that are less sensitive to dataset-specific variations.

Our experimental results demonstrate that a unified framework integrating of supervised contrastive learning, a dual-head architecture, and layer-wise normalization effectively enhances generalization across datasets. Supervised contrastive learning encourages ECG segments belonging to the same rhythm class to form compact and consistent groups in the feature space, regardless of their dataset origin. This property is particularly important in cross-dataset settings, where intra-class variability can otherwise dominate the learned representations. The dual-head architecture enables joint optimization of contrastive representation learning and rhythm classification, allowing the shared feature extractor to capture class-level relational structure while retaining discriminative power for binary rhythm classification. In addition, the layer-wise normalization strategy contributes to stabilizing cross-dataset performance by preserving global signal characteristics in early layers and reducing sensitivity to dataset-specific distribution shifts in deeper layers.

Overall, the proposed framework consistently outperformed single-dataset training and simple dataset-merging baselines under the LODO setting. The reduced performance variability across test datasets highlights the importance of learning dataset-invariant representations for reliable ECG classification in realistic deployment scenarios. These advancements support the development of more reliable AI tools that can maintain consistent performance in realistic clinical deployment scenarios.

## Figures and Tables

**Table 1:** Summary of the ECG datasets used in this study, including data source, number of records, and recording length.

Datasets	Country/Institution	#Records ^1^	Length (s) ^2^
Chapman–Shaoxing	China	45,152	10
CPSC 2018	China (CPSC 2018)	13,256	6~144
Georgia	USA (Southeastern US)	20,672	5~10
PTB	Germany (PhysioNet)	549	10~120
PTB-XL		21,837	10

^1^ # Records denotes number of records. ^2^ Recording length indicates the duration of the original ECG records provided in each dataset.

**Table 2:** Number of normal and AF samples used in the experiments for each dataset after preprocessing.

Database	Normal	AF	Total
Chapman–Shaoxing	1366	422	1788
CPSC 2018	1201	1266	2467
Georgia	1735	77	1812
PTB	928	60	988
PTB-XL	6432	35	6467

**Table 3:** Overall classification performance comparison across different training strategies. Overall results are presented as mean [95% confidence interval]. Detailed dataset-specific evaluation metrics and statistical significance tests are provided in Appendix C, Table A7 and Table A8, respectively.

Test Dataset	*Accuracy*			*Specificity*		
	Single	Baseline	Proposed	Single	Baseline	Proposed
Chapman–Shaoxing	87.6	82.4	98.4	100	99.0	99.9
CPSC 2018	69.4	93.4	94.4	37.1	86.4	93.6
Georgia	94.9	96.7	97.8	97.1	96.7	89.0
PTB	97.7	98.9	98.5	100	99.8	99.7
PTB-XL	97.0	95.3	98.6	97.0	95.3	98.6
Average	89.3 [74.6, 100]	93.3 [85.3, 100]	97.5 [95.3, 99.8]	86.2 [52.0, 100]	95.4 [88.8, 100]	96.2 [90.2, 100]
	***F1-Score***			***PR-AUC***		
	**Single**	**Baseline**	**Proposed**	**Single**	**Baseline**	**Proposed**
	71.8	66.3	97.7	89.8	84.7	99.8
	64.7	93.3	94.4	97.4	99.5	99.5
	70.3	84.7	88.6	68.2	86.2	86.8
	86.4	94.8	92.9	91.6	97.8	94.5
	67.2	58.0	72.9	56.0	82.3	83.2
	71.9 [61.1, 82.7]	79.5 [59.0, 99.9]	89.3 [77.2, 100]	80.6 [58.7, 100]	90.1 [80.2, 100]	92.8 [83.4, 100]

**Table 4:** Comparison of classification performance across different normalization strategies. Detailed evaluation metrics are provided in Appendix C, Table A9.

Normalization Strategy	*Accuracy*	*Specificity*	*F1-Score*	*PR-AUC*
Batch normalization	97.4	93.7	88.2	92.5
Instance normalization	88.2	98.7	75.6	95.2
Layer-wise normalization	97.5	96.2	89.3	92.8

**Table 5:** Comparison of classification performance for different loss function configurations. Detailed evaluation metrics are provided in Appendix C, Table A10.

$L_{SupCon}$	$L_{BCE}$	α	*Accuracy*	*Specificity*	*F1-Score*	*PR-AUC*
o	x	1	63.9	60.0	39.1	28.3
x	o	0	88.1	98.7	75.2	94.7
o	o	0.25	92.9	98.7	81.8	92.5
o	o	0.5	97.5	96.2	89.3	92.8

**Table 6:** Classification performance comparison of different multi-dataset training methods. Detailed evaluation metrics and statistical significance tests are provided in Appendix C, Table A11 and Table A12, respectively.

Method	*Accuracy*	*Specificity*	*F1-Score*	*PR-AUC*
Multi-dataset baseline	93.3	95.4	79.5	90.1
DANN	88.4	99.1	75.5	91.4
DSBN	97.2	98.1	86.3	92.9
MS-DANN	95.0	98.9	85.0	92.5
Proposed method	97.5	96.2	89.3	92.8

**Table 7:** Comparison of AF classification performance using multiple ECG datasets existing studies.

Works	Year	Datasets	# Leads ^1^	Length	*Accuracy*	*F1-Score*
Seo et al. [39]	2021	LTAF, AFDB, MITDB	2	10 s	86.5	-
Prabhakararao et al. [40]	2022	LTAF, AFDB, NSRDB	2	30 s	98.1	97.9
Liu et al. [41]	2022	AFDB, MITDB, NSRDB	1	32 RRIs	94.5	68.84
Zou et al. [42]	2024	CPSC 2021, LTAF, AFDB, MITDB, NSRDB	1	30 s	98.4	95.2
Toosi et al. [43]	2025	CinC 2017, LTAF, CPSC 2018, Private data	1	10 s	85.6	85.5
Proposed method	2026	Chapman–Shaoxing, CPSC 2018, Georgia, PTB, PTB-XL	1	10 s	97.5	89.3

^1^ # Leads denotes the number of leads used for training.

**Figure A1:**
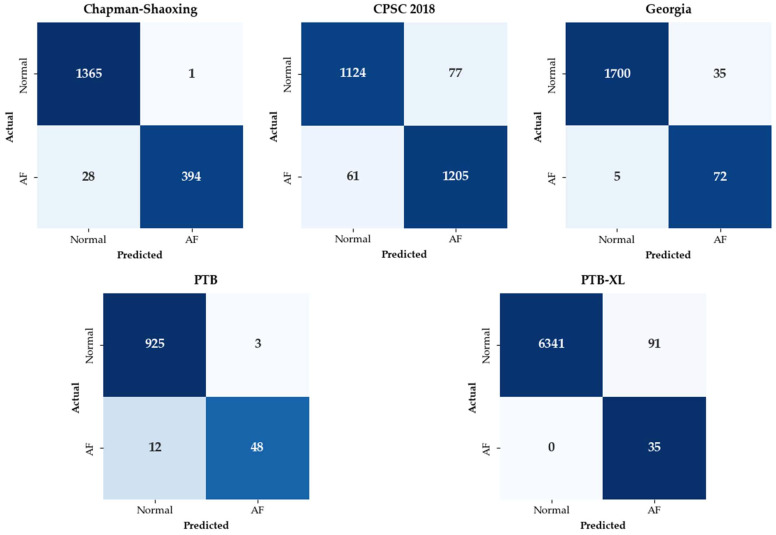
Confusion matrices of the proposed model across five datasets.

## Data Availability

The data presented in this study are openly available in PhysioNet. Reference number [29].

## Appendix A

### Detailed Model Architecture and Computational Resources

This appendix provides the technical specifications and structural details of the proposed model to ensure reproducibility. Table A1 summarizes the overall layer-by-layer configuration and functional components. The specific hyperparameters for all convolutional layers for each residual block are detailed in Table A2. Table A3 presents a comprehensive summary of the computational profiles.

**Table A1:** Structural specifications and functional components of the proposed model, including layer-wise output shapes, normalization types, and parameter counts.

Component	Stage/Layer	Output Shape	Normalization	Activation/Op	# Params ^1^
Input	Raw ECG	[1, 5000]	-	-	-
Feature extractor	Stem: Conv1d	[64, 2500]	BatchNorm	ReLU	640
	Stem: MaxPool1d	[64, 1250]	-	Downsampling	-
	Residual block 1-1	[64, 1250]	BatchNorm	Residual + ReLU	24,832
	Residual block 1-2	[64, 1250]	BatchNorm	Residual + ReLU	24,832
	Residual block 2-1	[128, 625]	BatchNorm	Residual + ReLU	62,016
	Residual block 2-2	[128, 625]	BatchNorm	Residual + ReLU	99,072
	Residual block 3-1	[256, 313]	InstanceNorm	Residual + ReLU	328,448
	Residual block 3-2	[256, 313]	InstanceNorm	Residual + ReLU	393,728
	Residual block 4-1	[512, 157]	InstanceNorm	Residual + ReLU	1,312,256
	Residual block 4-2	[512, 157]	InstanceNorm	Residual + ReLU	1,573,888
	Latent: GAP	[512, 1]	-	Avg pooling	-
	Latent: Flatten	[512]	-	-	-
	Latent: Dropout	[512]	-	*p* = 0.5	-
Projection head	Linear	[256]	-	ReLU	131,328
	Linear	[32]	-	-	8224
Classification head	Linear	[64]	-	ReLU	32,832
	Linear	[1]	-	Sigmoid	65
					Total: 4,013,473

^1^ # Params denotes the number of trainable parameters in the model.

**Table A2:** Detailed hyperparameters for all convolutional layers within the feature extractor, specifying kernel sizes (k), strides (s), and padding (p) for each residual block.

Stage/Block	Layer	Kernel (k)	Stride (s)	Padding (p)	Shortcut Path
Stem	Init Conv	7	2	3	-
Stage 1	Residual block 1-1	7/3	1/1	3/1	Identity
	Residual block 1-2	7/3	1/1	3/1	Identity
Stage 2	Residual block 2-1	7/3	2/1	3/1	1 × 1 Conv (s = 2)
	Residual block 2-2	3/3	1/1	1/1	Identity
Stage 3	Residual block 3-1	7/3	2/1	3/1	1 × 1 Conv (s = 2)
	Residual block 3-2	3/3	1/1	1/1	Identity
Stage 4	Residual block 4-1	7/3	2/1	3/1	1 × 1 Conv (s = 2)
	Residual block 4-2	3/3	1/1	1/1	Identity

**Table A3:** Computational efficiency and operational profiles of the proposed model.

Category	Value/Info
GPU hardware	NVIDIA GeForce RTX 4060 Ti
Software environment	PyTorch 2.5 with CUDA 12.1
Total parameters	4.01 M
Model size	36.57 MB
Training time (per epoch)	~12.0 s
Inference latency (per sample)	~3.5 ms

## Appendix B

### Rationale for Hyperparameter Selection

This section provides the empirical rationale for selecting the key hyperparameters through preliminary experiments to ensure optimal model performance. Table A4 summarizes the impact of the temperature parameter in the supervised contrastive loss. Table A5 illustrates the performance variations across different dimensions for the latent feature vector, while Table A6 details the sensitivity analysis for the projected embedding dimension used in the contrastive representation space.

**Table A4:** Comparison of classification performance for different temperature parameter (τ) in supervised contrastive loss.

Temperature (τ)	*Accuracy*	*Specificity*	*F1-Score*	*PR-AUC*
0.05	96.7	98.6	86.4	91.0
0.07	97.5	97.7	89.3	89.5
0.1	88.6	98.5	75.4	94.4
0.2	96.2	98.8	86.2	95.4

**Table A5:** Comparison of classification performance for different latent embedding (z) dimension.

Dimension	*Accuracy*	*Specificity*	*F1-Score*	*PR-AUC*
128	93.8	99.7	85.4	93.5
256	97.0	98.9	87.7	93.1
512	97.5	97.7	89.3	89.5
1024	97.4	99.0	87.5	95.0

**Table A6:** Comparison of classification performance for different latent embedding (h) dimension.

Dimension	*Accuracy*	*Specificity*	*F1-Score*	*PR-AUC*
16	88.7	99.4	79.8	85.8
32	97.5	97.7	89.3	89.5
64	95.2	99.1	87.5	88.7
128	97.6	98.9	88.5	95.2

## Appendix C

### Detailed Generalization Performance for ECG Classification

This section offers a detailed analysis of the experimental results to further validate the findings discussed in the main text. Table A7 summarizes the classification performance across five diverse test datasets for three different training strategies. Table A8 presents the results of statistical significance testing, including p-values from the Wilcoxon signed-rank test and effect sizes calculated via rank-biserial correlation. The localized performance and error distribution of the proposed model are visualized through confusion matrices for each test dataset in Figure A1.

**Table A7:** Detailed classification performance of each test dataset for the different training strategies.

Test Dataset		*Accuracy*			*Precision*			
		Single	Baseline	Proposed	Single	Baseline	Proposed	
Chapman–Shaoxing		87.6	82.4	98.4	100	90.2	99.7	
CPSC 2018		69.4	93.4	94.4	62.6	88.6	94.0	
Georgia		94.9	96.7	97.8	41.2	56.5	67.3	
PTB		97.7	98.9	98.5	100	96.2	94.1	
PTB-XL		97.0	95.3	98.6	15.3	10.1	27.8	
***Recall***			***Specificity***			***F1-Score***		
**Single**	**Baseline**	**Proposed**	**Single**	**Baseline**	**Proposed**	**Single**	**Baseline**	**Proposed**
47.4	28.4	93.4	100	99.0	99.9	71.8	66.3	97.7
100	100	95.2	37.1	86.4	93.6	64.7	93.3	94.4
45.5	96.1	93.5	97.1	96.7	89.0	70.3	84.7	88.6
61.7	85.0	80.0	100	99.8	99.7	86.4	94.8	92.9
100	97.1	100	97.0	95.3	98.6	67.2	58.0	72.9
***ROC-AUC***						***PR-AUC***		
**Single**		**Baseline**		**Proposed**		**Single**	**Baseline**	**Proposed**
96.1		95.5		100		89.8	84.7	99.8
97.8		99.3		99.5		97.4	99.5	99.5
93.8		98.0		97.7		68.2	86.2	86.8
98.8		99.8		98.9		91.6	97.8	94.5
94.1		99.7		94.1		99.7	58.0	72.9

**Table A8:** Statistical significance testing of performance improvements using the Wilcoxon signed-rank test and rank-biserial correlation to compare the proposed method against the baseline.

Metric	Proposed	Baseline	p-Value	Effect Size (r)
*Accuracy*	97.5	93.3	0.0625	0.867
*Specificity*	96.2	95.5	0.0625	0.867
*F1-score*	79.5	89.3	0.0938	0.733
*PR-AUC*	90.1	92.8	0.0938	0.733

Table A9 compares the classification performance of various normalization techniques to highlight the advantages of the proposed layer-wise strategy. Table A10 presents an ablation study on different loss function components and hyperparameter settings to validate the optimal training configuration. Table A11 evaluates the generalization capability of the proposed method against various domain generalization methods. Table A12 provides the results of the Wilcoxon signed-rank test and rank-biserial correlation to confirm the statistical significance of the performance improvements.

**Table A9:** Detailed classification performance for different normalization strategies.

Normalization Strategy	*Accuracy*	*Precision*	*Recall*	*Specificity*	*F1-Score*	*ROC-AUC*	*PR-AUC*
Batch normalization	97.4	72.8	93.7	98.7	88.2	99.2	92.5
Instance normalization	88.2	55.3	75.3	98.7	75.6	99.1	95.2
Layer-wise normalization	97.5	76.6	92.4	96.2	89.3	98.0	92.8

**Table A10:** Detailed classification performance for different loss function configurations.

$L_{SupCon}$	$L_{BCE}$	α	*Accuracy*	*Precision*	*Recall*	*Specificity*	*F1-Score*	*ROC-AUC*	*PR-AUC*
o	x	1	63.9	30.4	45.0	60.0	39.1	28.5	28.3
x	o	0	88.1	54.3	74.1	98.7	75.2	99.2	94.7
o	o	0.25	92.9	75.7	81.0	98.7	81.8	98.0	92.5
o	o	0.5	97.5	76.6	92.4	96.2	89.3	98.0	92.8

**Table A11:** Detailed classification performance for different multi-dataset training methods.

Method	*Accuracy*	*Precision*	*Recall*	*Specificity*	*F1-Score*	*ROC-AUC*	*PR-AUC*
Multi-dataset baseline	93.3	68.3	81.3	95.4	79.5	98.5	90.1
DANN	88.4	59.3	68.6	99.1	75.5	99.1	91.4
DSBN	97.2	71.2	93.0	98.1	86.3	99.2	92.9
MS-DANN	95.0	75.5	85.7	98.9	85.0	99.3	92.5
Proposed method	97.5	76.6	92.4	96.2	89.3	98.0	92.8

**Table A12:** Statistical significance testing of performance improvements using the Wilcoxon signed-rank test and rank-biserial correlation to compare the proposed method against various methods.

Method	*F1-Score*	p-Value	Effect Size (r)
Multi-dataset baseline	79.5	0.094	0.733
DANN	75.5	0.094	0.733
DSBN	86.3	0.313	0.333
MS-DANN	85.0	0.062	0.867
Proposed method	89.3	-	-
